# Supplementary material for: Comprehensive Taxonomical Analysis of Trichophyton mentagrophytes/interdigitale Complex of Human and Animal Origin from India
Source: J Fungi (Basel). 2023 May 16;9(5):577. doi: 10.3390/jof9050577 (PMC10219406; doi:10.3390/jof9050577)
Supplement: Supplementary file 1 [file jof-09-00577-s001.zip › jof-2350963-supplementary.pdf]

Supplementary Table S1: Primers used in the present study

Gene		5' to 3'	Reference
**Internal transcribed spacer**	Forward	TCCGTAGGTGAACCTTGCGG	Rudramurthy, AAC 2018
	Reverse	TCCTCCGCTTATTGATATGC	
**Translational elongation factor**	Forward	CACATTAACTTGGTCGTTATCG	deHoog Mycopatho 2017
	Reverse	CATCCTTGGAGATACCAGC	
**Beta tubulin**	Forward	GTBCACCTYCARACCGGYCARTG	deHoog Mycopatho 2017
	Reverse	CCRGAYTGRCCRAARACRAAGTTGTC	
**Large ribosomal subunit**	Forward	ACCCGCTGAACTTAAGC	deHoog Mycopatho 2017
	Reverse	TCCTGAGGGAAACTTCG	
**Calmodulin**	Forward	TGTCCGAGTACAAGGAAGC	Ahmadi, Medical mycology, 2016
	Reverse	TTACAATCAATTCTGCCGTC	
**High mobility group transcription factor**	Forward	AGGGAAACTTCAATTCCATCA	Tang Mycopatho 2021
	Reverse	GACAGCGTGAACAGAGTCTATC	
**α-box**	Forward	TCTCCTGCTGCCATGGGAGCT	Tang Mycopatho 2021
	Reverse	CCATTGGATTGATGTGTGCA	

Supplementary Table S2: The discrepancy in the naming of *Trichophyton mentagrophytes* complex in the NCBI database

Sl No.	Strain No.	Naming as per current study (ITS gene)	Naming in NCBI database ITS	Accession No. ITS	Naming in NCBI database TEF	Accession No TEF	Naming in NCBI database BT	Accession No. BT	Naming in NCBI database LSU	Accession No. LSU	Naming in NCBI database CAL	Accession No. CAL	Naming in NCBI database HMG	Accession No. HMG
**1**	V296-56	Ti			Tm	MW551676							Tm	MW551787
**2**	V296-58	Ti			Tm	MW551674							Tm	MW551785
**3**	CBS 428.63 NT (Ti)	Ti	Ti/Ti/Tm/Tm	JX122216/ NR_144900/ KT155896/MH858319										
**4**	ATCC 9533	Ti	Ti/Tm	KJ606115/ KC146353										
**5**	XM2	Ti											Tm	MW551681
**6**	XM12	Ti											Tm	MW551690
**7**	XM15	Ti											Tm	MW551692
**8**	XM16	Ti											Tm	MW551693

**9**	TSM 7	Ti	Tm	MK313070										
**10**	TSM 8	Ti	Tm	MK313072										
**11**	ZM 32	Ti	Tm	MK313074										
**12**	ZM 30	Ti	Tm	MK313073										
**13**	CBS 647.73 T (Ti)	Ti	Tm	KT155955			Tm	KT155595	Tm	KT155300				
**14**	CBS 475.93 T (Ti)	Ti	Tm/Ti	MH862433/ MF926357					Tm	MH874082 / MF893235				
**15**	IHEM 4411	Ti	Av	GU646873										
**16**	CBS 124425	Tm type III*	Ti	KT155763			Ti	KT155455	Ti	KT155090				
**17**	CBS 124421	Tm type III*	Ti	KT155761			Ti	KT155453	Ti	KT155088				
**18**	CBS 124420	Tm type III*	Ti	KT155760			Ti	KT155452	Ti	KT155087				
**19**	CBS 124415	Tm type III*	Ti	KT155758			Ti	KT155450	Ti	KT155085				
**20**	CBS 124410	Tm type III*	Ti	KT155756			Ti	KT155448	Ti	KT155083				
**21**	IHEM 19646	Tm type III*	Av	GU646874										
**22**	216520 /17	Tm type VIII	Av	GU646874										
**23**	VPCI 390/P/ 17	Tm type VIII	Ti	MH990852										
**24**	TSM 11	Tm type VIII	Ti	MK313042										
**25**	VPCI 2372/P /15	Tm type VIII	Ti	MH990809										
**26**	VPCI 387/P/ 17	Tm type VIII	Ti	MH990849										
**27**	VPCI 2146/P /16	Tm type VIII	Ti	MH990837										
**28**	VPCI 1242/P /16	Tm type VIII	Ti	MH990818										
**29**	VPCI 1239/P /16	Tm type VIII	Ti	MH990817										
**30**	VPCI 2150/P /16	Tm type VIII	Ti	MH990838										
**31**	VPCI 2054/P /16	Tm type VIII	Ti	MH990833										
**32**	VPCI 2033/P /16	Tm type VIII	Ti	MH990831										
**33**	VPCI 1068/P /15	Tm type VIII	Ti	MH990808										
**34**	VPCI 2382/P /16	Tm type VIII	Ti	MH990841										

**35**	VPCI 972/P/16	Tm type VIII	Ti	MH990814										
**36**	VPCI 233/P/16	Tm type VIII	Ti	MH990812										
**37**	VPCI 190/P/16	Tm type VIII	Ti	MH990810										
**38**	VPCI 822/P/15	Tm type VIII	Ti	MH990803										
**39**	VPCI 241/P/16	Tm type VIII	Ti	MH990813										
**40**	VPCI 220/P/16	Tm type VIII	Ti	MH990811										
**41**	VPCI 2038/P/16	Tm type VIII	Ti	MH990832										
**42**	VPCI 1407/P/16	Tm type VIII	Ti	MH990820										
**43**	VPCI 1984/P/16	Tm type VIII	Ti	MH990826										
**44**	VPCI 14/P/17	Tm type VIII	Ti	MH990844										
**45**	VPCI 2056/P/16	Tm type VIII	Ti	MH990834										
**46**	VPCI 924/P/15	Tm type VIII	Ti	MH990805										
**47**	VPCI 393/P/17	Tm type VIII	Ti	MH990853										
**48**	VPCI 2387/P/16	Tm type VIII	Ti	MH990843										
**49**	VPCI 2059/P/16	Tm type VIII	Ti	MH990835										
**50**	VPCI 2140/P/16	Tm type VIII	Ti	MH990836										
**51**	VPCI 1981/P/16	Tm type VIII	Ti	MH990824										
**52**	VPCI 2005/P/16	Tm type VIII	Ti	MH990829										
**53**	VPCI 1993/P/16	Tm type VIII	Ti	MH990827										
**54**	VPCI 1870/P/16	Tm type VIII	Ti	MH990822										

**55**	VPCI 1416/P/16	Tm type VIII	Ti	MH990821										
**56**	VPCI 976/P/15	Tm type VIII	Ti	MH990807										
**57**	VPCI 1261/P/16	Tm type VIII	Ti	MH990819										
**58**	VPCI 109/P/15	Tm type VIII	Ti	MH990801										
**59**	VPCI 1032/P/14	Tm type VIII	Ti	MH990799										
**60**	VPCI 953/P/15	Tm type VIII	Ti	MH990806										
**61**	VPCI 93/P/15	Tm type VIII	Ti	MH990800										
**62**	VPCI 389/P/17	Tm type VIII	Ti	MH990851										
**63**	VPCI 388/P/17	Tm type VIII	Ti	MH990850										
**64**	VPCI 386/P/17	Tm type VIII	Ti	MH990848										
**65**	VPCI 246/P/17	Tm type VIII	Ti	MH990846										
**66**	VPCI 2386/P/16	Tm type VIII	Ti	MH990842										
**67**	VPCI 2364/P/16	Tm type VIII	Ti	MH990840										
**68**	VPCI 230/P/17	Tm type VIII	Ti	MH990845										
**69**	VPCI 2156/P/16	Tm type VIII	Ti	MH990839										
**70**	VPCI 2025/P/16	Tm type VIII	Ti	MH990830										
**71**	VPCI 1983/P/16	Tm type VIII	Ti	MH990825										
**72**	VPCI 1979/P/16	Tm type VIII	Ti	MH990823										
**73**	VPCI 1163/P/16	Tm type VIII	Ti	MH990816										
**74**	VPCI 1083/P/16	Tm type VIII	Ti	MH990815										
**75**	NCCPF 800062	Tm type VIII	Ti	MH517560										

**76**	490	Tm type VIII	Ti	JN133999	Ti	KM678182	Ti	JF731044						
**77**	SRMC -K	Tm type VIII	Ti	KP099600										
**78**	Ahv-18	Tm type VIII	Ti	KT192468										
**79**	VPCI 2004/P/16	Tm type VIII	Ti	MH990828										
**80**	VPCI 383/P/17	Tm type VIII	Ti	MH990847										
**81**	VPCI 397/P/17	Tm type VIII	Ti	MH990854										
**82**	VPCI 747/P/15	Tm type VIII	Ti	MH990802										
**83**	VPCI 871/P/15	Tm type VIII	Ti	MH990804										
**84**	30816	Tm type VIII	Ti	MG889853										
**85**	CBS 646.73_ATCC 28145_Type (Tm)	Tm type IV	Tv/Av	MF926364/Z98014			Tv	MF898389	Tv	MF893245				
**86**	SJEK4836	Tm type IV	Ti	FM986773										
**87**	RV 27960	Tm type IV	Av	AF170452									Ti	AB605767
**88**	CCF<CZE>:4473	Tm type III*	Ti	LN736306										
**89**	IHEM 23029	Tm type III*	AV	GU646879										
**90**	AF506034	Tm type III	Ab	AF506034										
**91**	JCM 1891	Tm type IV			Ti	KM678058	Av	JF731120	Av	AB075330				
**92**	CBS 232.76	Ti	Tm	KT155838			Tm	KT155518	Tm	KT155177				
**93**	CBS 110.65	Ti	Tm	MH858507			Tm	KT155378	Tm	MH870141/KT155001				
**94**	CBS 559.66	Ti	Tm	MH858883/KT155943			Tm	KT155588	Tm	MH870542/KT155287				
**95**	CBS 113880	Ti	Tm	KT155711			Tm	KT155406	Tm	KT155030				
**96**	CBS 449.74	Ti	Tm	KT155904			Tm	KT155567.1	Tm	KT155249				
**97**	CBS 117723	Ti	Tm	KT155725			Tm	KT155418	Tm	KT155047				
**98**	CBS 119447	Ti	Tvio	KT155733			Tvio	KT155429	Tvio	KT155058				
**99**	CBS 124424	Tm type III*	Ti	KT155762			Ti	KT155454	Ti	KT155089				
**100**	CBS 124408	Tm type III*	Ti	KT155755					Ti	KT155082				

**101**	CBS 124401	Tm type III*	Tb	KT155753			Tb	KT155446	Tb	KT155080				
**102**	CBS 120357	Tm type III*					Ti	KT155438	Ti	KT155069				
**103**	CBS 120356	Tm type III*					Ti	KT155437	Ti	KT155068				
**104**	CBS 124426	Tm type III*	Ti	KT155764			Ti	KT155456	Ti	KT155091				
**105**	CBS 304.38 Type ™	Tm type IV	Ti	MF926360					Ti	MF893229				
**106**	2642	Tm type III	Ti	FM986750										
**107**	MASR	Tm type V	Tver	KU496915										
**108**	Ahv-331	Tm type VIII	Ti	KT192500										
**109**	RV 27961	Tm type IV	Av	AF170453										
**110**	KMU 5471	Tm*	Av	AB617775										
**111**	XQ3_8	Tm type IX	Ti	KU315316										
**112**	NBRC 5974	Ti	Tm			KM678206	Tm	JF731087						
**113**	CBS 435.73	Ti	Tm	MH860733					Tm	MH872444	Tm	KM387111		
**114**	CBS 126.34 Type (Tm)	Tm	Tm/Ti	MF926356/ AJ270790										
**115**	CBS 10 1546	Tq	Tm	JX122271	Tm						Tm	KM387113		
**116**	CBS 31 8.56	Tq	Tm/Ab	MH857656/KT155871/AY185126/Z97995	Tm	KM678083	Tm	KT155546/KU378223	Tm	MH869197/KT155215/KU205264	Tm	KM387110		

Tm- *Trichophyton mentagrophytes*, Ti- *Trichophyton interdigitale*, Tindo- *Trichophyton indotineae*, Av- *Arthroderma vanbreuseghemii*, Tv- *Trichophyton vanbreuseghemii*, Tb- *Trichophyton benhamiae*, Tver- *Trichophyton verrucosum*, Tvio- *Trichophyton violaceum*, Ab-*Arthroderma benhamiae*

Figures-

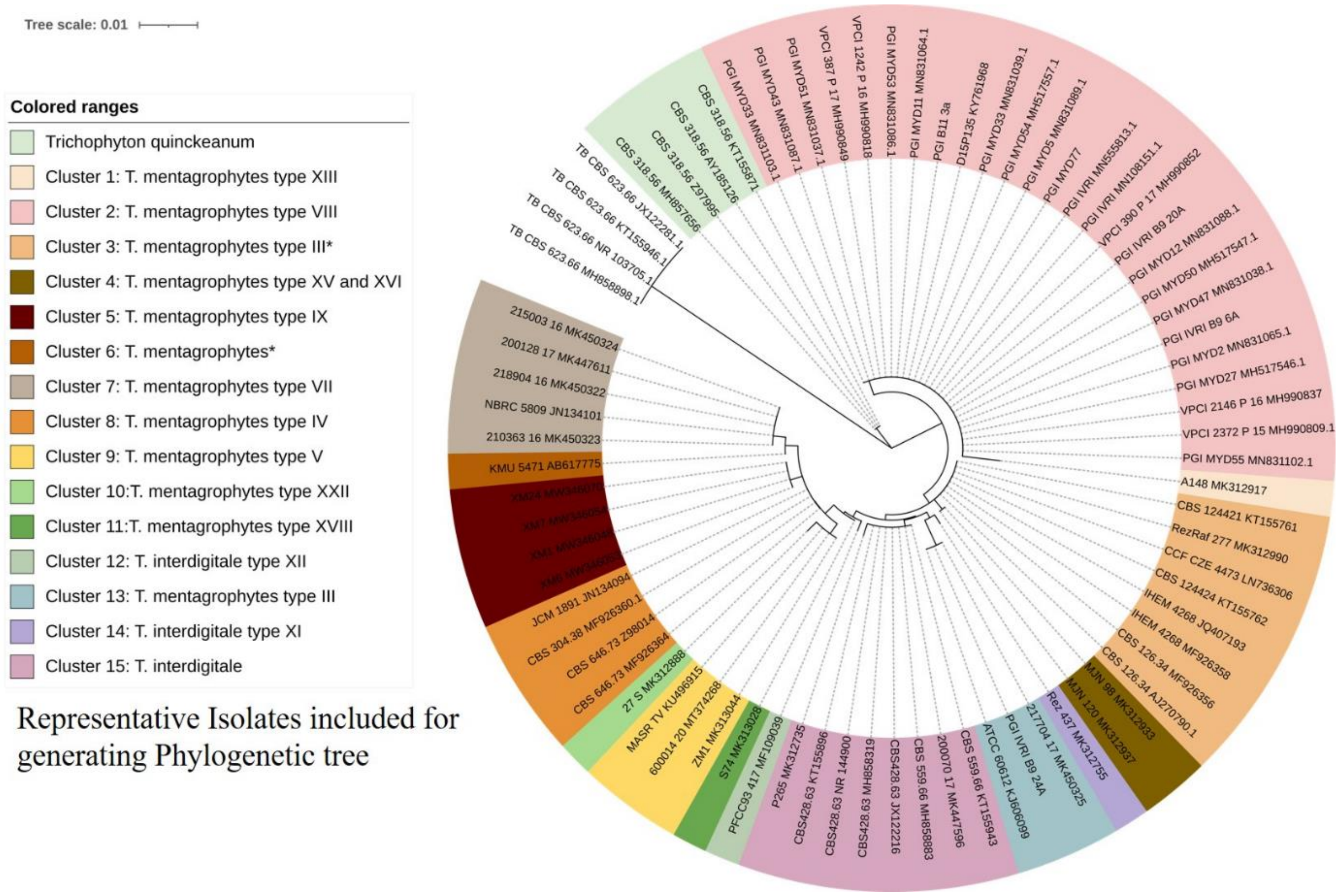

Supplementary Figure S1: Phylogenetic tree based on ITS sequences Neighbor-Joining method. The percentage of replicate trees in which the associated taxa clustered together in the bootstrap test (1000 replicates). The evolutionary distances were computed using the Kimura 2-parameter method. All positions containing gaps and missing data were eliminated. Evolutionary analyses were conducted in MEGA X.

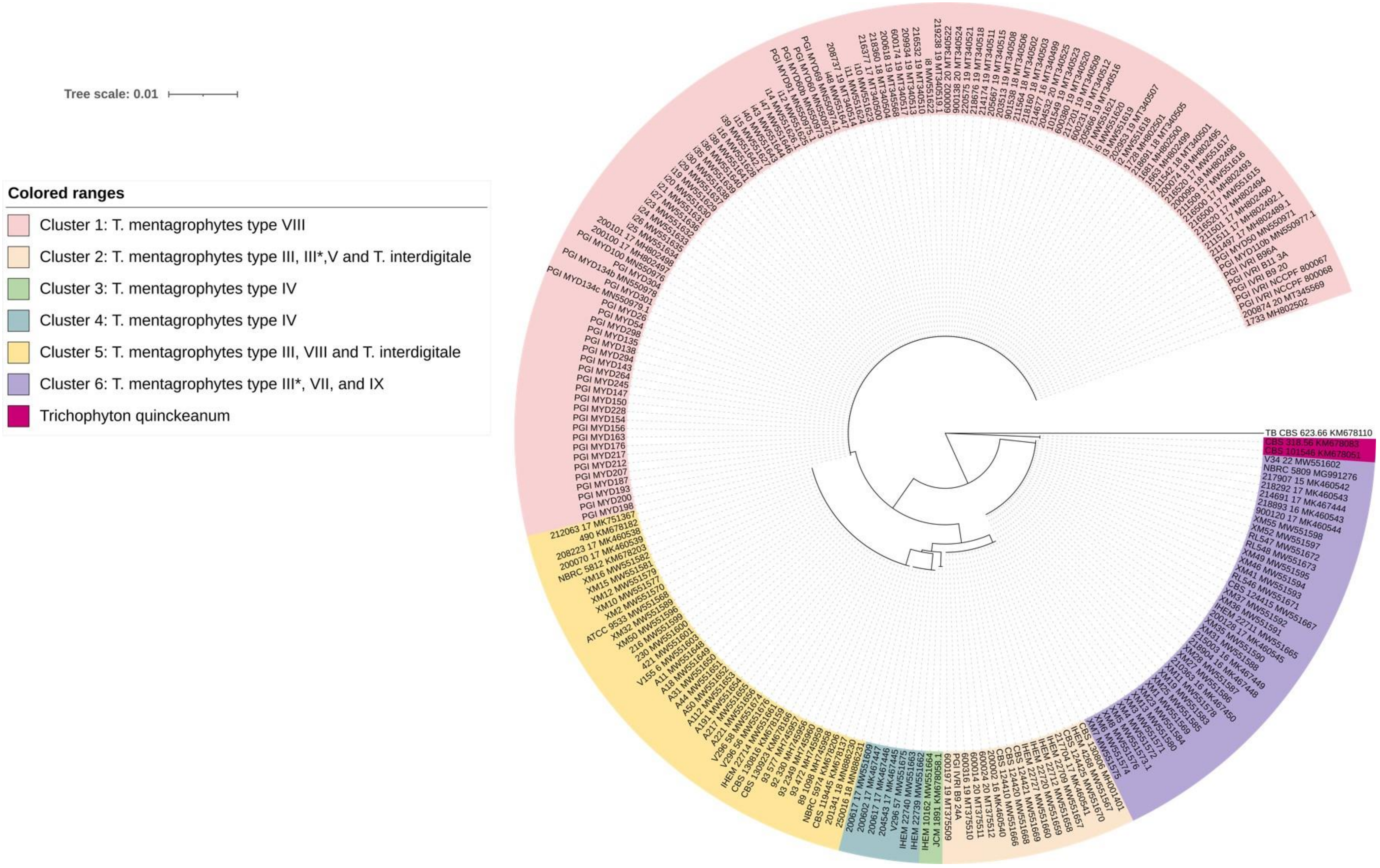

Supplementary Figure S2: Phylogenetic tree based on TEF sequences Neighbor-Joining method. The percentage of replicate trees in which the associated taxa clustered together in the bootstrap test (1000 replicates). The evolutionary distances were computed using the Kimura 2-parameter method. All positions containing gaps and missing data were eliminated. Evolutionary analyses were conducted in MEGA X.

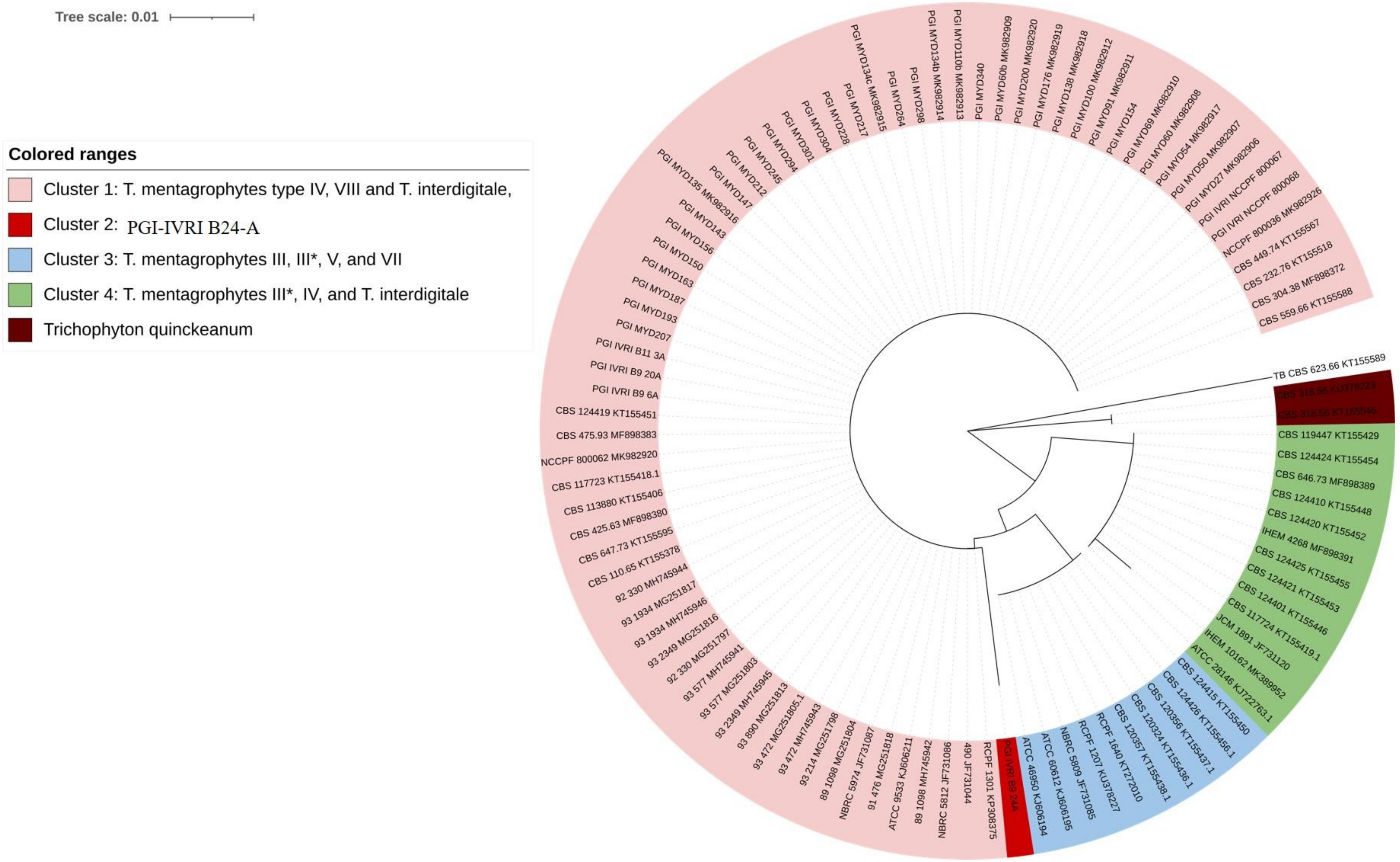

Supplementary Figure S3: Phylogenetic tree based on BT sequences Neighbor-Joining method. The percentage of replicate trees in which the associated taxa clustered together in the bootstrap test (1000 replicates). The evolutionary distances were computed using the Kimura 2-parameter method. All positions containing gaps and missing data were eliminated. Evolutionary analyses were conducted in MEGA X.

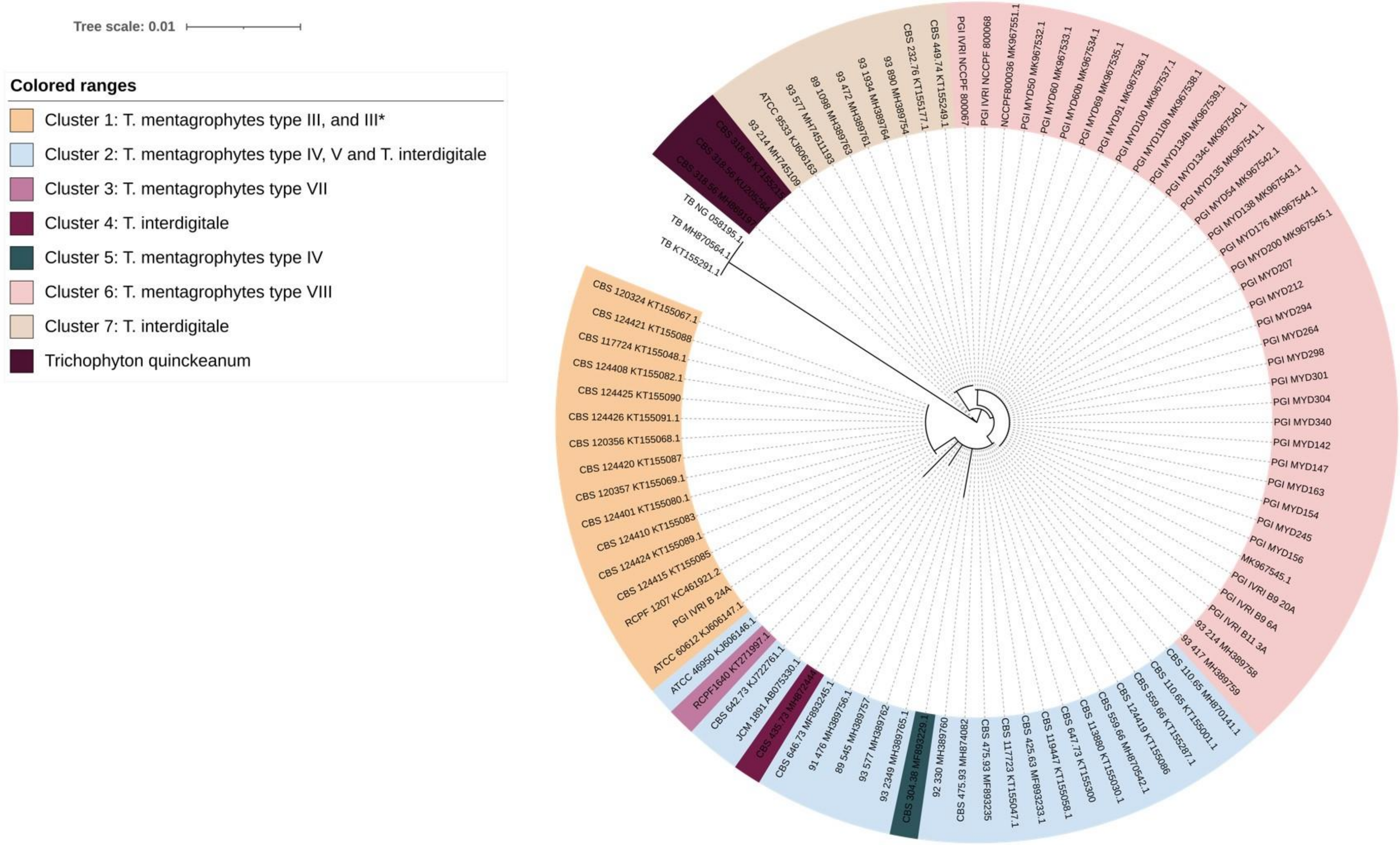

Supplementary Figure S4: Phylogenetic tree based on LSU sequences Neighbor-Joining method. The percentage of replicate trees in which the associated taxa clustered together in the bootstrap test (1000 replicates). The evolutionary distances were computed using the Kimura 2-parameter method. All positions containing gaps and missing data were eliminated. Evolutionary analyses were conducted in MEGA X.

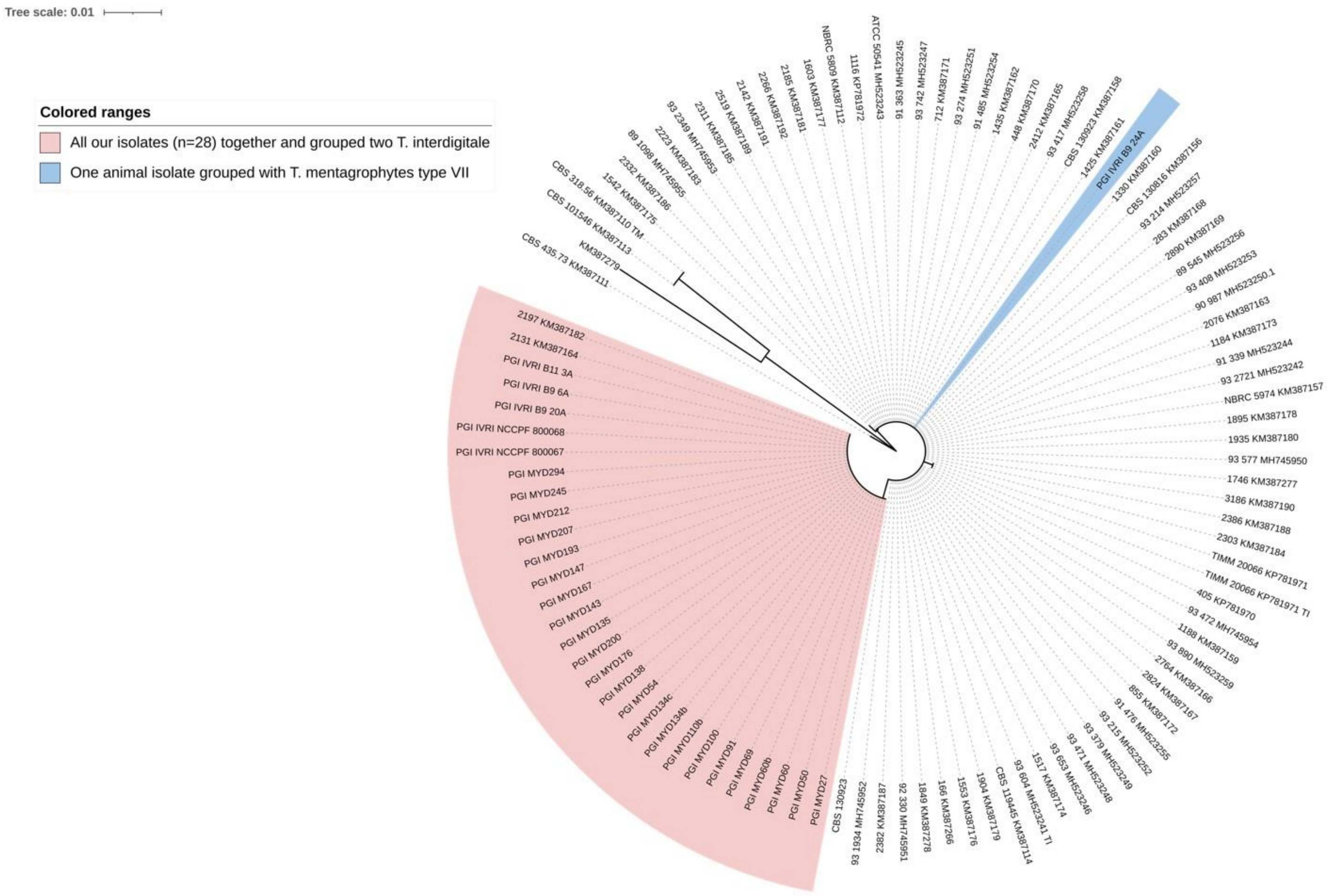

Supplementary Figure S5: Phylogenetic tree based on CAL sequences Neighbor-Joining method. The percentage of replicate trees in which the associated taxa clustered together in the bootstrap test (1000 replicates). The evolutionary distances were computed using the Kimura 2-parameter method. All positions containing gaps and missing data were eliminated. Evolutionary analyses were conducted in MEGA X.

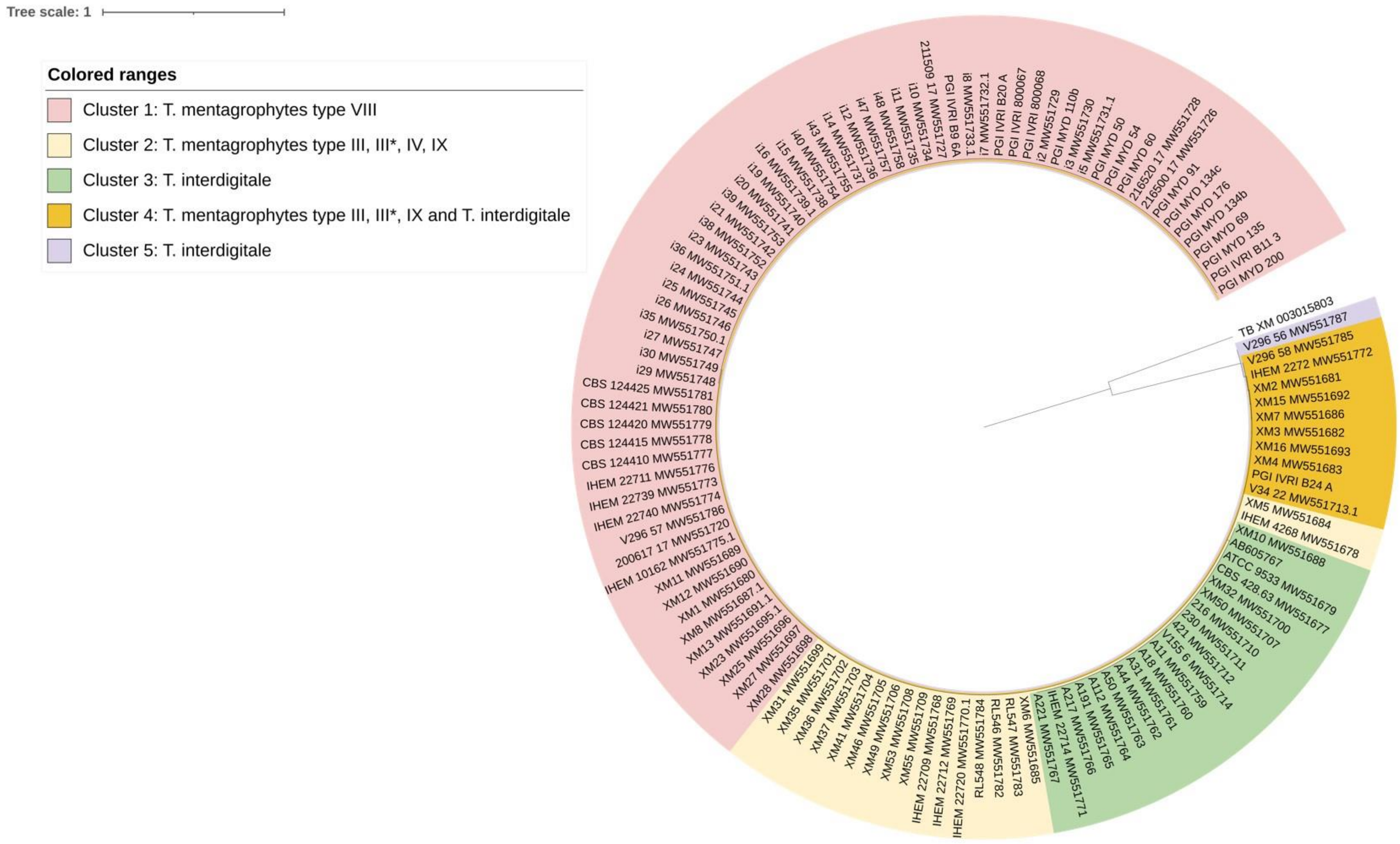

Supplementary Figure S6: Phylogenetic tree based on HMG sequences Neighbor-Joining method. The percentage of replicate trees in which the associated taxa clustered together in the bootstrap test (1000 replicates). The evolutionary distances were computed using the Kimura 2-parameter method. All positions containing gaps and missing data were eliminated. Evolutionary analyses were conducted in MEGA X
